# Battling COVID-19 in Bangladesh A Conversation with Dr. John Clemens of icddr,b

**DOI:** 10.4269/ajtmh.20-0504

**Published:** 2020-06-04

**Authors:** Claire Panosian Dunavan

**Affiliations:** 1 University of California, Los Angeles, California, E-mail: cpanosian@mednet.ucla.edu

On April 13, 2020, a New York Times editorial (“The Global COVID-19 Crisis is Poised to Get Much, Much Worse”) painted two sides of a planetary fault line. On one side were advanced economies girding themselves against COVID-19 and calculating how to weather the storm—on the other, poorer countries with far fewer tools to fight the pandemic. The final impression? A world deeply divided between nations with first-world “agency” and those that were passive and weighed down with dread.

The essay bared painful, obvious truths. Of course SARS-CoV-2 could exact a terrible toll on “countries ravaged by conflict, through packed refugee camps and detention centers in places like Syria or Bangladesh, through teeming cities like Mumbai, Rio de Janeiro, or Monrovia, where social distancing is impossible and government is not trusted…” to quote its authors.

And yet, I could not help but wince while reading the *Times* editors’ penultimate sentence, namely, “… the weakness of Washington should not prevent the brain trust of the developed world—the think tanks, news media, universities, and nongovernmental organizations—from focusing on a strategy for the next and possibly most brutal front in the struggle against the scourge of the COVID-19.” At first blush, who could disagree? At the same time, failing to acknowledge the skills and insights of other countries and entities seemed naive and paternalistic.

This brings us to Bangladesh. As a densely packed country of 161 million residents already burdened with natural and man-made challenges—infectious diseases, poor air quality, flooding, and refugees, for example, along with a “double epidemic” of diseases of poverty and development—the largely marshy delta crossed by 700 rivers could suffer devastating blows from COVID-19. At the same time, Bangladesh is a newly middle-income nation which has achieved major gains in child survival, reproductive health, and adult life span over the last several decades. Much of this success reflects its energized leaders, engaged citizens, and cadres of health workers with the potential to conduct the kind of grassroots public health that advanced economies have not yet envisioned, much less implemented, during the new global crisis.

Finally, Bangladesh is home to icddr,b (formerly, the International Centre for Diarrhoeal Disease Research, Bangladesh)—a one-of-a-kind, multinational institute now in its 60th year. The icddr,b’s current portfolio of research focused on improving the health of low- and middle-income countries spans bench to bedside and populations to health systems. What follows is an interview with Dr. John Clemens, who spent 5 years at icddr,b in the mid-1980s and returned there in 2013 as its executive director. For more highlights of Dr. Clemens’s career, please see the Addendum at the end of this piece.

## INTERVIEW WITH JOHN CLEMENS^†^

### Let’s start by comparing icddr,b in 2020 with the institution where you first worked. Can you paint a quick picture of how it has changed over time?

In the 1980s, the icddr,b focused primarily on diarrheal diseases as well as population studies and a bit on maternal–child health (MCH). By 2013, it had grown dramatically. It is now an organization of over 5,000 employees with a $70-million-a-year budget and a research agenda that extends well beyond diarrheal diseases to virtually all infectious diseases, maternal and child health, nutrition studies, reproductive health and rights, noncommunicable diseases, and adaptation to climate change. So, today, it’s a very broad-based institution. When I was at icddr,b in the 1980s, the microbiology laboratories could be best described as “Louis Pasteur-era,” with candle jars and the like. Now, we have super-modern laboratories which can bring molecular science to human populations, not only in the clinic but in the field.

### You’re currently running the institute from afar, which must be frustrating. Please share what makes icddr,b and Bangladesh so special to you.

Right now I am working from Seoul, as I had to leave Dhaka due to my age and other issues. I would have had to work from home in Dhaka had I not departed. So, probably on balance, I’m almost as effective here as I would be if I were there. But yes, it is hard not to be there right now. One of the reasons I’ve always loved Bangladesh and the center is that it’s where the rubber meets the road. You walk out of your residence in the morning and right there in the street you face the problems you’re addressing in your research projects. There’s a tragic abundance of disease and disability, but that of course provides tremendous opportunities for research that are not necessarily available elsewhere.

The main thing that allows my current working arrangement to succeed is our extraordinary team in Dhaka who put in 18- and 20-hour days and are really doing a yeoman’s job. Our national staff are incredibly dedicated; many folks devote their entire working lives to the institute. If you want to be a scientist in the medical and public health arena, it’s by far the best place in the country, and one of the best places in the developing world, to work.

As for Bangladesh itself, well, I think it has one of the warmest, most embracing cultures I’ve ever experienced. In addition, it’s an intellectual culture. The first Nobel Prize winner from the Indian subcontinent came from Bengal. And it’s a very hardworking, can-do culture. It’s a pleasure to work alongside Bangladeshi colleagues.

You have to think back to the 1970s when then-U.S. Secretary of State Henry Kissinger referred to Bangladesh as the world’s basket case. And in a certain sense, it was. Back then, it was East Pakistan—an agrarian backwater, densely populated, poor, beset by natural disasters and famine. And out of this emerged this country that is now very successful. A lot of credit goes to the Bangladeshi people.

### Let’s move on to COVID-19. When did you realize the immense threat it could pose to Bangladesh?

In the beginning, we all followed the events in Wuhan and didn’t know quite what to make of the risk outside China. It was obviously a potential risk, but it just wasn’t clear where things were going. What really shook me was the outbreak in Korea, which happened early on, and demonstrated how this virus can move. At that point, I realized that Bangladesh was going to be a tinderbox for the epidemic—not just as one of the most densely populated countries of the world but as a country with a double burden of disease. Not only does Bangladesh have traditional problems of developing countries like malnutrition, it now suffers from concurrent epidemics of diabetes, hypertension, and COPD [chronic obstructive pulmonary disease], all of which set people up for more severe COVID. And of course, the healthcare system is not strong.

### What’s the current situation in terms of mitigating COVID-19?

Bangladesh has been in lockdown since late March, and the lockdown will likely continue through May. The lockdown has been enforced by the police and the army. Of course, if you think about it, with the limited healthcare resources currently available in Bangladesh, social distancing will be Custer’s Last Stand until a vaccine becomes available.

### No question there. I’m just thinking of all the people who have to work to eat.

That’s our worry as well. Even though food is available in markets and stores if you have money, a huge proportion of the population relies on daily wages. How they’re managing, nobody really knows. There’s a sense that people living in urban slums, in particular, are experiencing significant problems. For starters, Bangladesh is one of the most densely populated countries in the world, with more than 1,000 people per square kilometer. But the slums, called *bustees*, have been growing like gangbusters over the past decade, and their population density is estimated to be eight times as high as the national average. If you visit these slums, it is not uncommon to find eight or 10 people living in a single room. What does the stay-at-home order mean in that context, in terms of implementability? But even beyond that, a huge percentage of residents—rickshaw drivers and the like—rely on daily wages. So there’s fear of huge collateral damage.

The one thing that could favor a better outcome for Bangladesh is the population age structure. Despite incredible gains in family planning and population control over the years, it’s still a young population that may well experience a lower mortality from COVID. However, it’s also possible that the outcome of treatment [i.e., lockdown] will be worse than the disease. In this regard, food and nutrition are a particular concern.

### Do you have a dedicated group at icddr,b that’s currently focused on COVID-19? If so, what are its principal activities?

Right now, it’s essentially an all hands on deck operation with respect to COVID. There is so much to do. The icddr,b is actively helping the government in testing and in tracing contacts. We have folks who are advising the government on high-level technical committees. The government has established new laboratories around the country for COVID diagnosis, and we’ve been training those laboratories on how to do PCR—not a minor task. And we’re training clinicians in other hospitals on intensive respiratory care, and we are working with UNICEF to create and run a dedicated COVID treatment center for the Rohingya refugees, in Teknaf, Bangladesh, where we have been working for years. As well, we are working with the government on field studies to better understand the transmission dynamics of COVID in Bangladesh, and we are collaborating with Bangladesh pharma on a study to evaluate locally produced drugs with demonstrable anti-COVID in vitro activity in the treatment of patients. As might be expected in light of our long track record in evaluating vaccines, we are also in discussions with several vaccine developers about conducting phase III trials.

We have a small but outstanding intensive care unit in our own hospital. It’s the place where icddr,b developed a new approach for childhood pneumonia called bubble continuous positive airway pressure (CPAP). We are now investigating how this can be adapted to adults with severe COVID, and possibly avert the need for mechanical ventilator support, a topic of very high importance to developing countries, where mechanical ventilators may not be readily available.

### Who are the principal funders for the clinical care you offer?

We operate three hospitals which together treat upwards of 200,000 patients per year, all free of charge. Most of these patients present with diarrhea, malnutrition, or acute respiratory infections, and our patient population is indigent. Beyond the government of Bangladesh, which partially funds the hospital, we basically find funds from indirect costs off of our grants and, to some extent, from our other core donors including Sweden, Canada, and the United Kingdom. But not all funders are comfortable with covering clinical services, so sometimes we have to get creative—robbing Peter to pay Paul so to speak.

### Let’s return to vaccine-related research at icddr,b. Given its history of testing vaccines for cholera and other infectious diseases, how likely is it that the institute will evaluate vaccines for SARS-CoV-2?

Because the COVID epidemic is significant already in Bangladesh, I believe there will be an opportunity to evaluate clinical protection as well as vaccine safety. The icddr,b is already a major international hub for vaccine trials. When COVID hit, we had 14 vaccine trials that were either ongoing or about to be launched for diseases as diverse as polio, influenza, typhoid, and hepatitis E. We had recently completed vaccine trials for RSV [respiratory syncytial virus], shigellosis, *Rotavirus*, enterotoxigenic *E. coli*, and cholera. And, these trials ranged from phase two to phase four. We have enormous expertise in executing trials that comply with modern international standards.

It’s obviously going to be important to understand how well SARS-CoV-2 vaccines work in developing country settings like Bangladesh where, because of population density, the force of infection will likely be very high, and, because of underlying comorbidities, the immune responses may not be ideal.

### Let’s also revisit icddr,b’s work on technologies to support patients with acute respiratory distress. Please fill in some details.

One of our scientists, Dr. Mohammod Chisti, working with colleagues at the University of Melbourne, adapted the old concept of CPAP—which has been used for decades in newborn intensive care units and is also used for sleep apnea—for the treatment of childhood pneumonia. In comparison with the WHO standard of low-flow oxygen for settings without ventilators—which is the case in most of the developing world—bubble CPAP reduced mortality by 75% in a head-to-head trial. Bubble CPAP is the epitome of appropriate and affordable technology, requiring only an oxygen source—which can be an oxygen tank—two plastic tubes to convey inspired oxygen and expired air, and a simple bottle—a soap bottle will do—that has a water level below which the end of the expiry tube is placed so that expired air meets calibrated resistance, creating bubbles and helping to open up alveoli. We have been very much involved, both in Bangladesh and beyond, in seeing whether bubble CPAP could be scaled up and effective in frontline district hospitals where ventilators don’t exist. And by the way, as of April there were only about 2,000 working ventilators in Bangladesh.

### How does the bubble CPAP approach work in adults?

Well, that’s the question. We’ve found that bubble CPAP operates basically on the delivery of oxygen and the expiry of air through nasal prongs. This works well in kiddies, but it doesn’t work quite so well in adults due to air leakage. Right now, Dr. Chisti is actively collaborating with an engineering group and using 3D printing to try to adapt the delivery system to adults. We are hoping that this will provide an effective approach for supporting adult COVID patients with severe respiratory illness.

### Switching gears, what can you share about the risk of COVID-19 in refugees in Bangladesh?

There is a large Rohingya population in southern Bangladesh in a district called Cox’s Bazar on the Bay of Bengal. This is a population that has experienced persecution in Myanmar and that has been entering Bangladesh for decades, and in a major way in August 2017, when nearly a million streamed in from Myanmar. They are currently occupying a huge swath of camps in that area.

When they first arrived, they were in an acute, desperate situation complicated by torrential rains and no shelter, no clean water, no sanitation, and no food. The Bangladesh government, NGOs, and the international community really rose to the occasion. The vulnerability of the Rohingyas was tragically illustrated by a huge epidemic of diphtheria due to very low vaccine coverage of Rohingya children in Myanmar and densely populated, severe living conditions in the camps. An example of the wonderful collaboration between the government and relief agencies was the recognition of the high risk of cholera when the Rohingyas arrived, and prompt, preemptive delivery of oral cholera vaccine from the global stockpile, which averted a near-certain epidemic. Dr. Firdausi Qadri and her team from icddr,b played a critical role in this important effort.

The situation has stabilized since their mass ingress into Bangladesh in 2017, and although COVID cases have not been reported among the Rohingyas to date,^‡^ this population is clearly at high risk of a significant COVID outbreak because the camps are very densely populated and have limited healthcare facilities equipped to care for acutely ill people. And so, the same agencies that have been active since 2017 remain active. The icddr,b has participated not only in the oral cholera vaccine campaigns but in operating diarrhea treatment centers and malnutrition rehabilitation units. Now UNICEF is coordinating agencies including icddr,b, Médecins sans Frontieres, Save the Children, the Red Cross, and others to set up treatment centers for COVID for this population. As we speak, those facilities are being constructed and we’re in the process of staffing them.

### Any thoughts about COVID-19’s negative impact on other public health priorities?

Obviously, diverting personnel and resources from preventing and caring for other conditions is taking a toll, although we don’t yet have enough data to estimate the toll. This was well documented for maternal mortality during the recent Ebola epidemic in West Africa. And, just as in that Ebola outbreak, COVID seems to be fueling widespread reluctance in the population to come to hospitals for fear of contracting the infection. However, other factors may also be at play. Because Bangladesh has been on lockdown since late March—and despite efforts by the government and NGOs to provide food to the poor—we know that many people are suffering. A recent, still-unpublished survey by icddr,b of both rural and urban populations documented high levels of food insecurity in about 50% of households surveyed during the lockdown. Some authors speculate that the major burden of morbidity and mortality in developing countries during the COVID pandemic will come from such lockdowns rather than from COVID itself.

### As a final note, what lessons from Bangladesh might help other countries battling COVID-19?

A few years ago, there was a wonderful 3-part series in the *Lancet* on the Bangladesh paradox which basically asked the question: How has this country which was impoverished, densely populated, and experiences frequent natural calamities like typhoons been able to succeed both in health and development? In health, Bangladesh has seen tremendous gains in reduction of infant, child, and maternal mortality; economically—for years prior to COVID—the country grew at 6–7% per year and is now a middle-income country.

In large part, the government’s willingness to base public health policies on scientific evidence—much of it generated by icddr,b—explains Bangladesh’s success in health. A country-wide deployment of female community health workers who deliver oral rehydration solution packets, micronutrients, and family-planning services, based on the findings of a large MCH experiment done by icddr,b, has greatly aided the approach. Finally, the government and NGO sectors work hand in hand to implement successful public health strategies.

In the future, I believe that these uniquely integrated parties will help control COVID by continuing to work together through Bangladesh’s extensive network of community-based programs.

## Footnotes

† This interview was conducted by phone and email in April and May 2020, and later edited for clarity and length.

‡ On May 15, 2020, the first SARS-CoV-2 infection was detected in the Rohingya camps in Cox’s Bazar.

## Addendum:

*Remember aerograms? When John Clemens was a medical student at Yale, receiving those thin blue, folded letters from a college friend in the Peace Corps in Liberia awakened his interest in exotic travel. Two years later, with support from a Reader’s Digest fellowship, Clemens took a year’s leave of absence from Yale to work at a Mennonite hospital in Tanzania. “Maybe I can make more of an impact doing public health in developing countries than practicing medicine in the U.S.” he concluded after witnessing the hospital’s daily toll of life-threatening diarrhea—a grim fallout from the then-President Julius Nyerere’s policies which forcibly displaced many Tanzanians into agrarian cooperatives utterly devoid of clean water and sanitation*.

*Then, after finishing medical school at Yale, Clemens trained in internal medicine at Case Western Reserve, and, in 1978, made his first 6-week trip to the Southeast Asian Treaty Organization Cholera Research Laboratory (CRL) in Dhaka, a U.S. Cold War entity staffed by young physician-scientists from the NIH and CDC which would later become icddr,b. That career-changing trip was made possible by mentors Chuck Carpenter and Tom Butler at Case Western, and Bucky Greenough, scientific director of CRL, who was willing to take the young resident on. In 1983, with further epidemiologic training under his belt, Clemens returned to the then icddr,b, where Greenough had become the director, for the first five years of a three-decade effort to develop, test, and manufacture a low-cost oral cholera vaccine that is now widely used in developing countries and stockpiled by the WHO*.

*Before returning to Dhaka in 2013, Clemens’s previous positions included chief of epidemiology at the Center for Vaccine Development of the University of Maryland; chief of the epidemiology branch of the National Institute of Child Health and Human Development; founding director-general of the International Vaccine Institute in Seoul, Korea; and professor and vice chair of epidemiology at the UCLA School of Public Health*.

*In 2010, Clemens received the Sabin Gold Medal for his work on oral cholera vaccine. In 2017, he accepted the $2 million Hilton Humanitarian Award on behalf of icddr,b. In 2018, Clemens and Professor Jan R. Holmgren, of Sweden were honored with the Prince Mahidol Award for Public Health in recognition of their longstanding collaboration on oral cholera vaccine*.

## References

[b1] Chisti MJ 2015 Bubble continuous positive airway pressure for children with severe pneumonia and hypoxaemia in Bangladesh: an open, randomized controlled trial. Lancet 386: 1057–1065. 26296950 10.1016/S0140-6736(15)60249-5

[b2] Chowdury MR Bhuiya A Chowdhury ME Rasheed S Hussain Z Chen LC, 2013 The Bangladesh paradox: exceptional health achievement despite economic poverty. Lancet 382: 1734–1745. 24268002 10.1016/S0140-6736(13)62148-0

[b3] https://www.nytimes.com/2020/04/13/opinion/coronavirus-cases.html.

